# *OsIPK2* Acts as an Organ-Specific Modulator of Rice Trichome Development by Coordinating Cuticular Wax Metabolism and Transcriptional Regulation

**DOI:** 10.3390/plants15091414

**Published:** 2026-05-06

**Authors:** Yao Chen, Zhiqun Li, Mengyang Huang, Ninghan Shi, Yonghui Li, Kongyang Wu, Yanwei Cheng, Xuhao Liu, Sihong Sang

**Affiliations:** 1 College of Life Sciences, Luoyang Normal University, Luoyang 471934, China; 2 School of Pharmacy, Guizhou University of Traditional Chinese Medicine, Guiyang 550025, China

**Keywords:** trichome development, cuticular wax metabolism, *OsIPK2*, transcriptional regulation, rice (*Oryza sativa* L.), *Arabidopsis*

## Abstract

Trichomes are specialized epidermal structures that play pivotal roles in plant defense against biotic and abiotic stresses. Inositol polyphosphate kinase 2 (IPK2) is a key enzyme in inositol phosphate metabolism with diverse functions in eukaryotic cellular processes. However, its involvement in trichome development remains uncharacterized. Here, we systematically analyzed the function of a rice inositol polyphosphate kinase gene (*OsIPK2*) in trichome development using transgenic rice lines and heterologously expressing *Arabidopsis* lines. Scanning electron microscopy (SEM) analysis revealed that *OsIPK2* acts as an organ-specific modulator of trichome development in rice. Its overexpression repressed macrohair initiation and microhair elongation in leaves, while promoting trichome development on the glumes. Metabolomic profiling revealed that *OsIPK2* overexpression reprogrammed cuticular wax metabolism in transgenic rice leaves, shifting fatty acid flux toward long-chain wax precursors and increasing soluble carbohydrate levels. Transcriptomic and qPCR analysis confirmed that *OsIPK2* modulated the expression of genes involved in cuticular wax biosynthesis, auxin homeostasis, and the core trichome regulatory cascade in rice. Conversely, heterologous overexpression of *OsIPK2* in *Arabidopsis* strongly suppressed trichome initiation and branching, resulting in drastically reduced trichome density and fewer trichome branches. These phenotypes were associated with the downregulation of the MYB-bHLH-WD40 (MBW) transcriptional complex and its downstream target genes. Collectively, our findings suggest that *OsIPK2* modulated trichome development through organ- and species-specific mechanisms. In rice, it coordinated wax metabolism and the OsSPL10-OsSCR1/2-OsWOX3B-OsHL6 cascade to affect organ-specific trichome formation. In *Arabidopsis*, it inhibited trichome development by repressing the MBW complex. These results uncover a novel role of *OsIPK2* in plant epidermal cell fate specification and advance our understanding of the molecular mechanisms underlying organ- and species-specific regulation of trichome development.

## 1. Introduction

Trichomes are specialized epidermal structures widely distributed on the aerial surfaces of terrestrial plants. As physical barriers, trichomes help modulate surface temperature, reduce water transpiration, enhance tolerance to UV radiation, and improve resistance to herbivores [1]. Given the significance of trichomes in crop growth and stress adaptation, it is critical to elucidate the regulatory mechanisms underlying trichome development in model plants, particularly in the monocot rice (*Oryza sativa* L.) and dicot *Arabidopsis thaliana* [2].

The regulatory network of trichome development has been well-documented in *Arabidopsis*. Trichome initiation and morphogenesis are mainly controlled by the MYB–bHLH–WD40 (MBW) transcriptional complex [3]. The R2R3-MYB transcription factor GL1, redundant bHLH factors GL3 and EGL3, and WD40-repeat protein TTG1 assemble into a stable core complex, which directly activates the downstream transcription factors GL2 and TTG2 to promote trichome formation. Mutations in any of these core components lead to glabrous or defective trichome phenotypes [4,5,6,7,8]. In contrast, several R3-MYB proteins compete with GL1 to bind GL3/EGL3, thus disrupting MBW complex assembly and repressing trichome initiation, including TRIPTYCHON (TRY), CAPRICE (CPC), and ENHANCER OF TRY, as well as CPC1 (ETC1), ETC2, ETC3, TRICHOMELESS1 (TCL1), and TCL2 [9,10,11]. These trichome regulators also participate in other epidermal physiological processes, such as anthocyanin biosynthesis, seed coat development, and root hair formation [12,13].

In contrast, the molecular mechanisms governing epidermal hair development remain largely elusive in rice. To date, only a few regulators have been identified, including *OsSPL10*, *OsSCR1/2*, *OsWOX3B*, and *OsHL6* [14,15,16]. These factors form a sequential regulatory cascade, OsSPL10-OsSCR1/2-OsWOX3B-OsHL6. OsSPL10, an SBP-box transcription factor, acts as a positive regulator of rice trichome initiation [15,17]. The GRAS-family SCARECROW proteins OsSCR1 and OsSCR2 function redundantly to promote trichome initiation, and the *osscr1 osscr2* double mutant exhibits a nearly glabrous phenotype [16]. *OsWOX3B* (also named *NUDA/GL-1*, *GLR1*, *LSY*) encodes a WOX3 family transcription factor required for the formation of macrohairs and microhairs, and its loss-of-function results in complete glabrous leaves and glumes [14,18]. *OsHL6* (*OsGL6*), an AP2/ERF transcription factor, positively modulates both the initiation and elongation of macrohairs [19]. At the regulatory level, OsSPL10 enhances the expression of *OsSCR1*, and the OsSPL10-OsSCR1 complex further induces the transcription of *OsWOX3B* to promote the differentiation of protodermal cells (PTCs) into trichome precursor cells (TPCs) [16]. OsSPL10 then interacts with OsWOX3B to modulate *OsHL6* expression, facilitating TPC elongation and trichome maturation, while OsHL6 physically associates with OsWOX3B to co-regulate macrohair initiation and elongation. Despite these findings, the upstream regulatory signals, metabolic basis, and organ-specific mechanisms underlying rice trichome development remain largely unknown.

Cuticular wax biosynthesis is tightly interconnected with trichome development [20]. Across diverse plant species, mutants defective in cuticle and wax biosynthesis often exhibit abnormal trichome morphology, indicating shared regulatory networks [21]. In *Arabidopsis*, wax biosynthetic mutants, such as *kcr1*, *gh6/cer10*, *yre/cer3*, and *abcg11*, show reduced trichome size or defective trichome structure, linking epicuticular wax metabolism to trichome morphogenesis [22,23,24,25]. In peaches, *PpMYB25* and *PpMYB26* coordinately regulate trichome initiation and cuticular wax accumulation on fruit skin. Loss-of-function of *PpMYB25* suppresses *PpMYB26*, causing both loss of trichomes and reduced wax, yielding smooth, glossy fruit [26]. In tomato, the *sticky peel* (*pe/CD2*) mutant displays reduced wax disposition and decreased glandular trichome density [27]. In rice, multiple genes such as *ACL1*, *OsMYB60*, and *OsGL1-6* enhance VLCFA-derived wax accumulation and improve drought tolerance [28,29,30]. However, the genetic link between wax accumulation and trichome formation remains unclear in rice.

Inositol polyphosphate kinase 2 (IPK2) is a conserved key enzyme in inositol phosphate metabolism [31]. *Arabidopsis* harbors two *IPK2* homologous genes (*AtIPK2α* and *AtIPK2β*), whereas rice contains only one (*OsIPK2*) [32]. Our previous studies have demonstrated that *OsIPK2* acts as a negative regulator in gibberellin (GA)-mediated shoot elongation and reproductive fertility in rice [33]. Additionally, *OsIPK2* functions as a chaperone protein for *OsIAA11*, a member of the AUX/IAA repressor, to stabilize it and suppress auxin-dependent lateral root development [34]. Recently, we demonstrated that *OsIPK2* is involved in Pi-mediated root development and is essential for seed germination and early seedling establishment [35,36]. However, its role in epidermal cell fate and trichome development has not been reported.

In this study, we characterized the function of *OsIPK2* in trichome development. We showed that *OsIPK2* modulated trichome development in an organ-specific manner. Its overexpression repressed macrohair initiation and microhair elongation in rice flag leaves, but promoted trichome formation on the glumes. Wax metabolomic analysis revealed that cuticular wax metabolism was reprogrammed in the OE-1 line, along with transcriptional changes in the core trichome-regulatory network in rice. Furthermore, heterologous expression of *OsIPK2* caused severe trichome-deficient phenotypes and suppressed the core MBW complex in *Arabidopsis*. Our findings suggest that *OsIPK2* is a novel modulator of trichome development by coordinating transcriptional and metabolic pathways, providing new insights into regulatory mechanisms of species- and organ-specific epidermal cell development in plants.

## 2. Results

### 2.1. OsIPK2 Modulates Trichome Development in Rice in an Organ-Specific Manner

In rice, two distinct non-glandular trichome types are typically present on leaves, macrohairs and microhairs [15]. Macrohairs specifically develop on silica cells overlying thin vascular bundles, whereas microhairs form alongside stomatal cells or adjacent to motor cells [19]. To reveal the function of rice inositol polyphosphate kinase gene (*OsIPK2*) in epidermal cell differentiation and trichome development, we used *OsIPK2* overexpression (OE) and RNA interference (Ri) transgenic rice lines, which have been characterized in our previous works [35,36]. We then performed scanning electron microscopy (SEM) to observe the morphology and distribution of epidermal hairs on both leaf blades and glumes of WT and transgenic lines.

**Figure 1:**
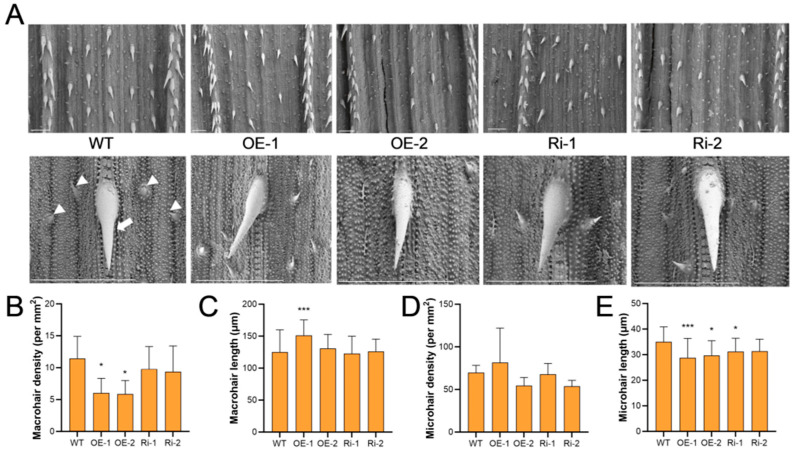
Trichome phenotype in rice leaves of *OsIPK2* overexpression and RNAi lines. (**A**) Scanning electron microscopy (SEM) images of macrohairs and microhairs on the leaf blades of WT, *OsIPK2-Ov* (OE-1, OE-2), and *OsIPK2-RNAi* (Ri-1, Ri-2) transgenic rice lines. The upper images show overall views of leaf trichomes, and the bottom images show close-up views of macrohairs and microhairs. Scale bar = 200 μm. (**B**,**C**) Macrohair density (**B**) and length (**C**) in WT and transgenic rice lines. Data represent mean ± SD (6 ≤ *n* ≤ 10). (**D**,**E**) Microhair density (**D**) and length (**E**) in WT and transgenic rice lines. Data represent mean ± SD (18 ≤ *n* ≤ 30). Asterisks represent significant differences compared with the corresponding WT by Student’s *t*-test (* *p* < 0.05, *** *p* < 0.001).

On the leaf blades, overexpression of *OsIPK2* exerted a prominent inhibitory effect on trichome development (Figure 1A). The density of macrohairs was reduced to 52.7% and 51.1% of wild-type (WT) levels in OE-1 and OE-2 lines, respectively (Figure 1B). In contrast, RNA interference of *OsIPK2* did not cause a significant change in macrohair density compared with WT. Notably, macrohair length was significantly increased by 20.8% in OE-1 compared with WT (Figure 1C), indicating that *OsIPK2* differentially modulated macrohair initiation and elongation. For microhairs, there were no significant differences in microhair density between WT and transgenic lines (Figure 1D). Microhair length in OE-1, OE-2, and Ri-1 lines was reduced to 82.2%, 84.8, and 89.2% relative to WT, respectively (Figure 1E). These results collectively indicate that *OsIPK2* modulated trichome development in rice leaves, mainly by repressing macrohair initiation and microhair elongation. Observation of leaf cross-sections showed that OE-1 exhibited a curled morphology, shrunken motor cells, reduced vascular bundle size, and sparse mesophyll cells, resulting in a disorganized and thinner leaf structure (Supplemental Figure S1). These impairments in epidermal cell specification and cell expansion likely disrupt the initiation and elongation of leaf trichomes.

**Figure 2:**
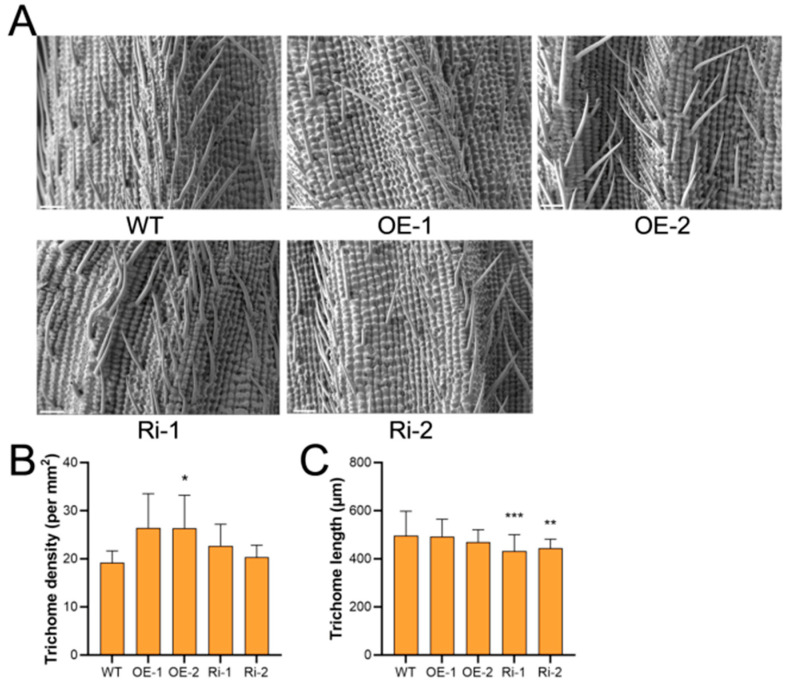
Trichome phenotypes on the glumes of *OsIPK2* overexpression and RNAi lines. (**A**) Scanning electron microscopy (SEM) images of trichomes on the glumes of WT, *OsIPK2-Ov* (OE-1, OE-2), and *OsIPK2-RNAi* (Ri-1, Ri-2) transgenic rice lines. Scale bar = 200 μm. (**B**) Trichome density of WT and transgenic rice lines. Data are means ± SD (6 ≤ *n* ≤ 7). (**C**) Trichome length of WT and transgenic rice lines. Data are means ± SD (*n* = 34). * *p* < 0.05, ** *p* < 0.01, *** *p* < 0.001 (Student’s *t*-test).

On the contrary, *OsIPK2* exhibited an opposing regulatory pattern for trichome development on the glumes (Figure 2A). SEM observation showed that the trichome density of the OE-2 line was increased 1.4-fold relative to WT (Figure 2B). Meanwhile, trichome length of Ri lines decreased to 87.1% and 89.5% of WT levels, but no significant change was observed in the OE lines (Figure 2C). These data suggest that *OsIPK2* acts as an organ-specific modulator of epidermal trichome development in rice. It repressed trichome initiation and elongation in leaves, yet promoted trichome formation on glumes, indicating that distinct regulatory mechanisms govern epidermal cell differentiation in these two tissues.

### 2.2. OsIPK2 Is Involved in Cuticular Wax Metabolism in Rice

**Figure 3:**
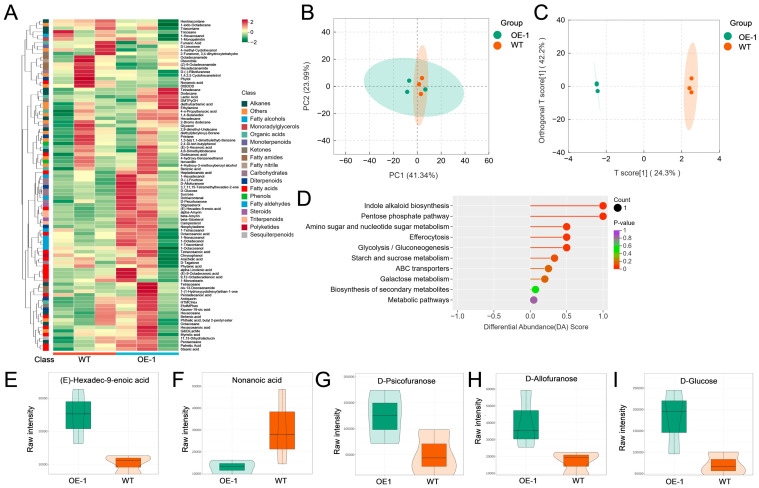
Targeted metabolomic profiling of cuticular wax-related metabolites on leaves of wild-type (WT) and *OsIPK2*-overexpressing (OE-1) transgenic plants. (**A**) Heatmap of identified wax-related metabolites in OE-1 and WT. Each column shows the normalized abundance of metabolites in one replicate. Class indicates the category of each wax-related metabolite. The color scale represents relative metabolite abundance after z-score normalization. (**B**) PCA score plot of OE-1 and WT. (**C**) OPLS-DA score plot of OE-1 and WT. (**D**) KEGG enrichment analysis of differentially abundant metabolites (DAMs). (**E**–**I**) Relative abundance of DAMs in WT and OE-1, including (E)-Hexadec-9-enoic acid (**E**), Nonanoic acid (**F**), D-Psicofuranose (**G**), D-Allofuranose (**H**), and D-Glucose (**I**).

Cutin and cuticular wax together form a dynamic, hydrophobic barrier over aerial epidermal cells that is essential for plants to defend against diverse environmental stresses [37]. Trichome formation is tightly coupled with cuticular wax biosynthesis in these epidermal cells. To elucidate the metabolic precursors underlying trichome development, we performed targeted metabolomic profiling of cuticular wax on leaves of WT and OE-1 plants (Figure 3; Supplemental Table S2). As shown in the classification pie chart, a total of 19 metabolite classes were detected in rice leaf samples (Supplemental Figure S2). Among them, fatty acids (18.48%) and alkanes (13.04%) were the two most dominant classes, followed by carbohydrates (8.70%), fatty alcohols (7.61%), diterpenoids (6.52%), organic acids (6.52%), and fatty amides (4.35%). Hierarchical clustering heatmap revealed a distinct metabolic signature between OE-1 and WT, with prominent differences in lipid-related compounds and carbohydrates (Figure 3A). Principal component analysis (PCA) showed excellent intra-group reproducibility of metabolic profiles from WT and OE-1 rice leaves (Figure 3B). It suggests that *OsIPK2* overexpression did not cause a global metabolic disturbance. Orthogonal partial least squares discriminant analysis (OPLS-DA) displayed complete separation between the two groups, confirming significant metabolic reprogramming induced by *OsIPK2* overexpression in rice leaves (Figure 3C). KEGG enrichment of differential abundant metabolites (DAMs) showed that the differential metabolites were significantly enriched in pathways associated with cuticular wax biosynthesis and carbon allocation. The enriched pathways include starch and sucrose metabolism, glycolysis and gluconeogenesis, the pentose phosphate pathway, and ABC transporters (Figure 3D).

A total of five metabolites (VIP > 1, |Log_2_(Fold Change)| ≥ 1) were identified as DAMs from 93 wax-related metabolites initially detected by GC-MS targeted analysis. In OE-1, nonanoic acid (medium-chain fatty acid) was downregulated to 43% of the WT level compared with WT, while (E)-Hexadec-9-enoic acid (long-chain precursor) was upregulated 2.4-fold relative to WT (Figure 3E,F). The contents of D-Psicofuranose, D-Allofuranose, and D-Glucose in the OE-1 line increased 2.4-, 2.4-, and 2.5-fold relative to WT, respectively (Figure 3G–I). Correlation analysis of DAMs revealed a strong positive correlation between these soluble carbohydrates and (E)-Hexadec-9-enoic acid (r ≥ 0.77) (Supplemental Figure S3). In contrast, nonanoic acid was negatively correlated with (E)-Hexadec-9-enoic acid (r = −0.63). It indicates a redirection of metabolic flux from medium-chain fatty acid synthesis to long-chain wax precursor production upon *OsIPK2* overexpression in transgenic rice leaves.

Collectively, these results demonstrate that *OsIPK2* is involved in metabolic flux in rice carbohydrate and lipid metabolism. The reduced trichome number in *OsIPK2*-overexpressing lines, together with the altered wax-related metabolite profiles, suggests a potential link between *OsIPK2*-associated metabolic reprogramming and epidermal cell fate specification.

### 2.3. Transcriptomic and qPCR Analysis of OsIPK2 Overexpression Plants

**Figure 4:**
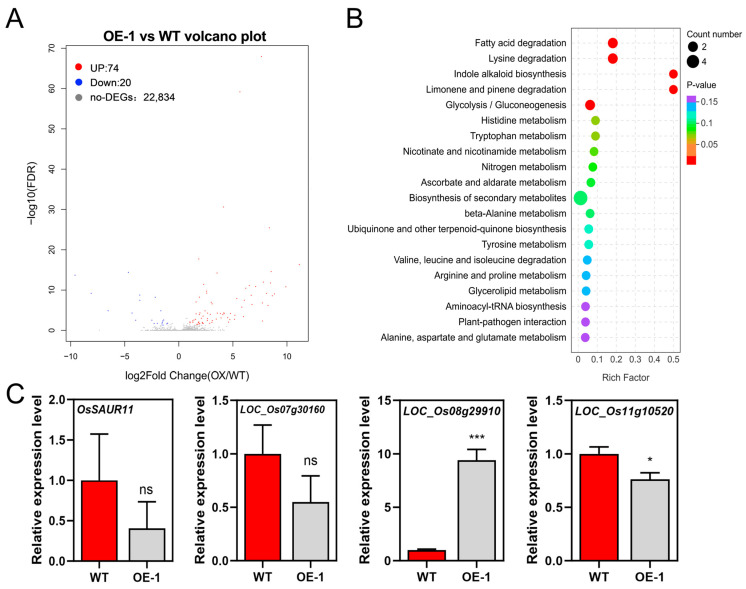
Transcriptomic analysis of wild-type (WT) and *OsIPK2* overexpression (OE-1) rice leaves. (**A**) GO enrichment analysis of differentially expressed genes (DEGs) between wild-type (WT) and OE-1. (**B**) KEGG enrichment analysis of DEGs. (**C**) qRT-PCR analysis of *OsSAUR11*, *LOC_Os07g30160*, *LOC_Os08g29910*, and *LOC_Os11g10520* in WT and OE-1. *OsACT2* was used as an internal control. Data are means ± SD (*n* = 3). * *p* < 0.05, *** *p* < 0.001 (Student’s *t*-test). ns indicates non-significance.

To understand the transcriptional regulatory network driving the metabolic shifts and epidermal development defects in *OsIPK2* transgenic rice, we performed transcriptome sequencing on the leaves of OE-1 and WT plants (Supplemental Table S3). There was a total of 94 annotated differentially expressed genes (DEGs) (|log_2_FoldChange| ≥ 1.00 and FDR < 0.05), including 74 upregulated DEGs and 20 downregulated DEGs (Figure 4A). KEGG pathway enrichment analysis revealed that pathways associated with trichome development were significantly enriched, including biosynthesis of secondary metabolites, glycolysis/gluconeogenesis, fatty acid degradation, and indole alkaloid biosynthesis (Figure 4B). These pathways generate the essential carbon backbones, energy, and structural components necessary for cuticular wax synthesis, polarized cell growth, and secondary metabolite accumulation. Genes involved in fatty acid and wax metabolism were significantly downregulated in OE-1 (Figure 4C), such as putative alcohol dehydrogenase (LOC_Os11g10520) and prenyltransferase (LOC_Os08g29910). In addition, *OsSAUR11* and a putative trehalose phosphatase were also significantly expressed in OE-1 compared with WT. Taken together, the transcriptomic data demonstrate that the overexpression of *OsIPK2* affected the transcription of genes involved in cuticular wax biosynthesis, auxin homeostasis, and carbon allocation.

**Figure 5:**
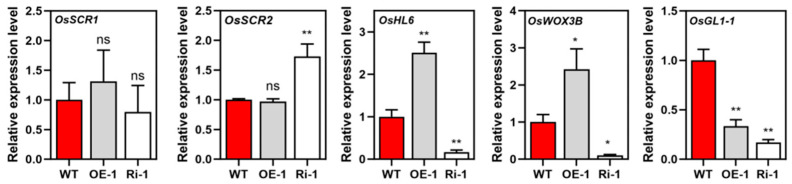
Expression analysis of trichome and wax-related genes in wild-type (WT), OE-1, and Ri-1 lines. qRT-PCR analysis of *OsSCR1*, *OsSCR2*, *OsHL6*, *OsWOX3B*, and *OsGL1-1* in WT, OE-1, and Ri-1 plants. *OsACT2* was used as an internal control. Data are means ± SD (*n* = 3). * *p* < 0.05, ** *p* < 0.01 (Student’s *t*-test). ns indicates non-significance.

To validate the expression changes in core trichome regulatory genes identified by transcriptomic analysis (Supplemental Figure S4), we performed quantitative real-time PCR (qRT-PCR) on key components of the OsSPL10-OsSCR1/2-OsWOX3B-OsHL6 cascade (Figure 5). Compared with WT, transcript levels of *OsSCR1* and *OsSCR2* in OE-1 showed no significant differences, while *OsSCR2* was significantly upregulated in Ri-1. In contrast, *OsHL6* and *OsWOX3B* were upregulated 2.5- and 2.4-fold in OE-1 relative to WT, respectively, but downregulated to 16.5% and 10% of WT levels in Ri-1. Notably, the transcript of *OsWOX3B* was not detected in our transcriptome dataset (no valid reads mapped in WT or OE-1 samples), likely due to low expression and below the reliable detection limit of RNA-seq. Meanwhile, the wax biosynthesis gene *OsGL1-1* was downregulated to 33% and 16.8% of WT levels in OE-1 and Ri-1 lines, consistent with the transcriptomic result. These qPCR results validated the transcriptomic data and revealed that *OsIPK2* modulates the core trichome regulatory module at the transcriptional level.

### 2.4. Ectopic Expression of OsIPK2 in Arabidopsis Suppressed Trichome Initiation and Branching

**Figure 6:**
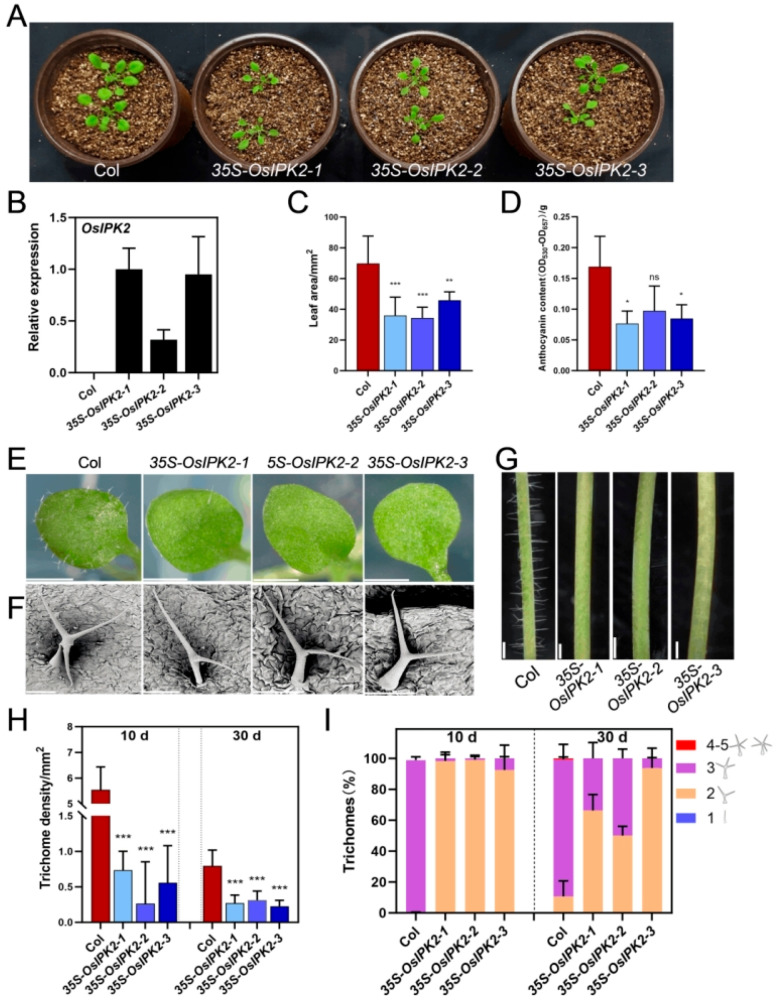
Phenotypic characterization of *35S-OsIPK2* transgenic *Arabidopsis* lines. (**A**) Growth phenotype of 3-week-old Col (WT) and *35S-OsIPK2* transgenic lines. Scale bar = 1 cm. (**B**) Relative expression of *OsIPK2* in Col and *35S-OsIPK2* lines by qRT-PCR. *OsUBQ5* was used as an internal control. Data shown are the mean ± SD of three replicates. (**C**) Leaf area of Col and *35S-OsIPK2* lines. Data are means ± SD (5 ≤ *n* ≤ 7). (**D**) Anthocyanin content of leaves from Col and *35S-OsIPK2* lines. Data are means ± SD (*n* = 3). (**E**) Representative images of rosette leaf trichomes from Col and *35S-OsIPK2* lines. Scale bar = 1 mm. (**F**) SEM images of leaf trichomes. Scale bar = 100 μm. (**G**) Phenotypes of stem trichome from Col and *35S-OsIPK2* lines. Scale bar = 1 mm. (**H**) Trichome density on rosette leaves of Col and *35S-OsIPK2* lines at 10 and 30 d. Data are means ± SD (5 ≤ *n* ≤ 7). (**I**) Proportions of trichomes with different branch numbers at 10 and 30 d. Data are means ± SD (5 ≤ *n* ≤ 7). * *p* < 0.05, ** *p* < 0.01, *** *p* < 0.001 (Student’s *t*-test). ns, non-significance.

Trichome morphogenesis and distribution are highly divergent between monocots and dicots. Unlike rice, *Arabidopsis* leaf trichomes are single-celled and branched. This morphological divergence implies distinct regulatory networks underlying trichome development in the two lineages. To assess whether the trichome-regulatory function of *OsIPK2* is conserved between rice and *Arabidopsis*, we obtained three independent homozygous T_3_ transgenic *Arabidopsis* lines *(35S-OsIPK2-1*, *-2*, and *-3*) constitutively overexpressing *OsIPK2* under the control of *CaMV 35S* promoter [35]. The transcription level of *OsIPK2* in these lines was validated by qRT-PCR (Figure 6A,B).

Compared to WT (Col) plants, all three *35S*-*OsIPK2* lines exhibited significantly reduced leaf area, with decreases of 48.6%, 50.7%, and 34.3%, respectively. They also showed reduced anthocyanin content, with decreases of 57.3%, 31.2%, and 49.9%, respectively (Figure 6C,D). Notably, 10 d old *35S-OsIPK2* lines displayed near-complete loss of trichomes on leaves (Figure 6E–G). These results indicate that constitutive overexpression of *OsIPK2* impaired leaf expansion and epidermal trichome development in transgenic *Arabidopsis*. Leaf cross-sectional analysis revealed that epidermal cells of *35S-OsIPK2* lines were more compactly arranged with thicker cell walls and irregular, wavy outlines, suggesting defects in cuticular wax deposition or cell wall integrity (Supplemental Figure S5).

The morphology of trichomes on the third leaves of 10 d old Col and *35S-OsIPK2* lines was characterized by diameter, branch length, and branch position (Table 1). The first branch lengths (the longest trichome branch) of transgenic lines were 89.2%, 79.8%, and 86.7% of Col levels, respectively. The second branch lengths in transgenic lines were 84.8%, 74%, and 70.8% of Col levels, respectively. The interbranch zone length (branch position relative to the trichome base) was significantly increased in *35S-OsIPK2-1* and *35S-OsIPK2-2* lines. Furthermore, the maximum diameters of the trichome cell bases in the *35S-OsIPK2* lines were all significantly reduced compared with that of Col.

As overexpression of *OsIPK2* suppressed leaf expansion, we examined the effect of *OsIPK2* on trichome initiation and branching in seedlings (10 d) and adult plants (30 d). We found that trichome densities on both the third leaves and rosette leaves were significantly decreased in transgenic lines (Figure 6H). Moreover, all transgenic lines had no trichome on the main inflorescence stems (Figure 6G). Quantification analysis showed that more than 99% of trichomes in the third leaves of Col plants were three-branched, while 92.5–99.2% of trichomes in transgenic lines were two-branched (Figure 6I). In rosette leaves of 30 d old transgenic plants, 51.3–93.8% of trichomes were two-branched. In contrast, 88.6% of trichomes of Col plants were three-branched. Taken together, these results demonstrate that overexpression of *OsIPK2* severely inhibited the initiation and branching of trichomes in transgenic *Arabidopsis* leaves.

*35S-OsIPK2* transgenic *Arabidopsis* plants exhibited growth-stunted phenotypes similar to auxin-resistant mutants such as *iaa16*, *iaa7*, and *ARF7-RNAi* transgenic plants (Supplemental Figure S6). Considering this, we crossed the *DR5-GFP* transgenic line with *35S-OsIPK2-1* to determine auxin distribution and accumulation (Supplemental Figure S7). In the Col background, strong *DR5-GFP* fluorescence was mainly observed in the quiescent center and columella cells. In contrast, the GFP signal was nearly absent in the root tip of *35S-OsIPK2-1*/*DR5-GFP* plants, indicating that *OsIPK2* attenuated auxin signaling in transgenic *Arabidopsis*. However, exogenous auxin treatment failed to rescue the trichome-defective phenotype in *35S-OsIPK2* lines (Supplemental Figure S8). Moreover, overexpression of the *Arabidopsis* homolog *AtIPK2β* did not trigger trichome defects in transgenic *Arabidopsis* leaves (Supplemental Figure S9) [38]. Together, these results suggest that *OsIPK2* did not inhibit trichome formation primarily by repressing auxin signaling or through the conserved inositol phosphate metabolism pathway in *Arabidopsis*.

### 2.5. Expression of OsIPK2 Repressed the MBW Transcriptional Complex in Transgenic Arabidopsis

**Figure 7:**
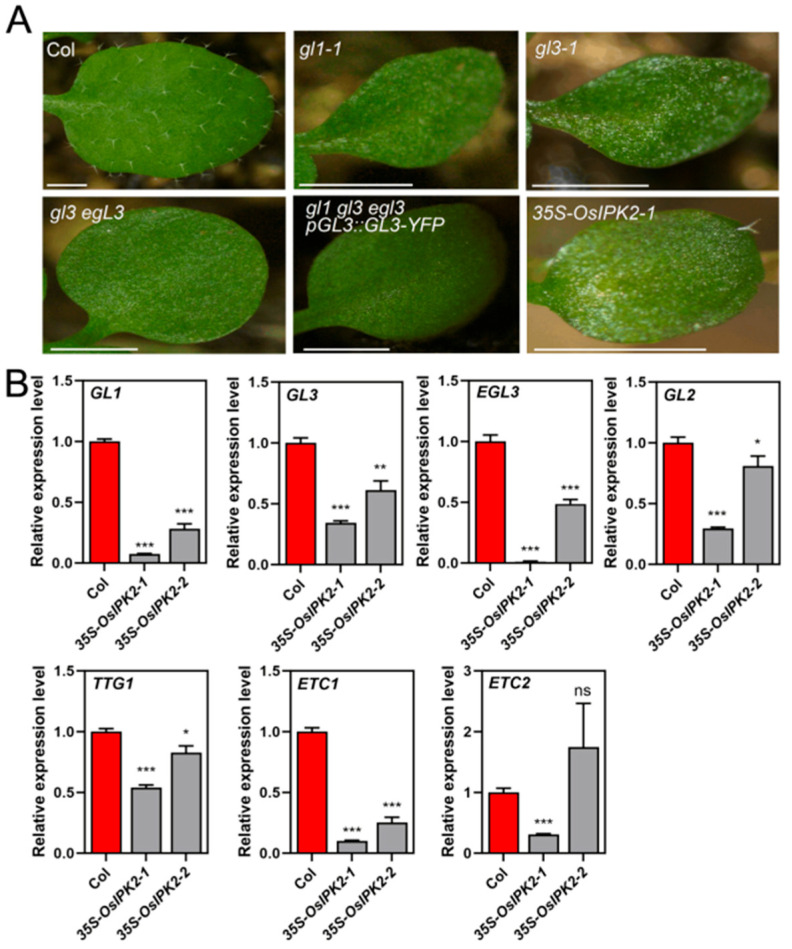
Expression analysis of trichome regulatory genes in *35S-OsIPK2* transgenic *Arabidopsis* lines. (**A**) Leaves of trichomeless mutants and *35S-OsIPK2* transgenic *Arabidopsis* plants. Scale bar = 1 mm. (**B**) qRT-PCR analysis of trichome regulatory genes in Col and *35S-OsIPK2* lines, including *GL1*, *GL3*, *EGL3*, *GL2*, *TTG1*, *ETC1*, and *ETC2*. The expression of *ACT8* was used as an internal control. Data are means ± SD (*n* = 3). * *p* < 0.05, ** *p* < 0.01, *** *p* < 0.001 (Student’s *t*-test). ns indicates non-significance.

In *Arabidopsis*, trichome initiation and morphogenesis are governed by the conserved MYB-bHLH-WD40 (MBW) transcriptional complex [5,39,40]. Phenotypic observation showed that the trichome defects in *35S-OsIPK2* overexpression transgenic *Arabidopsis* lines were highly similar to those of the glabrous mutants *gl1-1*, *gl3-1*, and *gl3 egl3*, yet with a milder effect (Figure 7A). To reveal the molecular mechanism underlying these trichome defects, we performed qRT-PCR to examine the transcript levels of core trichome regulatory genes. As shown in Figure 7B, the expressions of MBW complex components *GL1*, *GL3*, *EGL3* and the downstream target *GL2* were all significantly downregulated in *35S-OsIPK2* lines compared with Col. The expression of *TTG1* was significantly reduced in *35S-OsIPK2-1*, while partially restored in *35S-OsIPK2-2*. Notably, the expression of R3-MYB repressor genes *ETC1* and *ETC2* was also downregulated in *35S-OsIPK2* lines, consistent with their role as direct downstream targets of the MBW complex. We further performed semi-quantitative RT-PCR to detect the expression of additional trichome repressor genes *TRY*, *CPC* and *TCL1* (Supplemental Figure S10). Consistent with the qRT-PCR results, *GL1* and *GL2* transcript levels were markedly lower in *35S-OsIPK2* lines relative to Col. In contrast, the expression levels of *CPC*, *TRY* and *TCL1* were comparable between transgenic lines and WT plants.

Taken together, these findings demonstrate that *OsIPK2* impaired trichome development by repressing the core MBW transcriptional module, rather than by activating trichome repressor genes.

## 3. Discussion

Trichome formation and spatial patterning are critical agronomic traits in crop plants [2]. However, unlike *Arabidopsis*, only a few regulators controlling trichome development have been characterized in rice, and the molecular regulatory networks remain largely unclear. Our previous work revealed the functions of *OsIPK2* in seed germination, lateral root formation, shoot elongation, reproductive fertility, and phosphate homeostasis [33,35,36]. In this study, we further uncovered an organ-specific regulatory function of *OsIPK2* in trichome development in rice.

### 3.1. OsIPK2 Modulates Organ-Specific Trichome Development in Rice

The OsSPL10-OsSCR1/2-OsWOX3B-OsHL6 pathway constitutes the core regulatory cascade governing trichome formation in rice. OsSPL10 directly activates *OsHL6* transcription, while *OsSCR1/2* redundantly enhance *OsWOX3B* expression [15,16]. OsWOX3B physically interacts with OsHL6 to boost its DNA-binding affinity for auxin-related genes, such as *OsYUCCA5* and *OsPIN1b*, thereby promoting auxin accumulation and polar transport to drive trichome initiation and elongation [19]. Loss-of-function mutations in *OsHL6* or *OsWOX3B* led to glabrous leaves and glumes [18,41].

In this study, qPCR analysis revealed that *OsIPK2* positively regulates this core regulatory cascade (Figure 5). Compared to WT, the transcript levels of *OsWOX3B* and *OsHL6* were upregulated 2.4- and 2.5-fold in *OsIPK2*-overexpressing (OE) lines, while *OsSCR1* and *OsSCR2* expression remained unchanged. Conversely, *OsHL6*, *OsWOX3B*, and *OsGL1*-*1* were significantly downregulated in *OsIPK2*-*Ri* lines (Figure 1 and Figure 2). Notably, in OE lines, the activated OsWOX3B-OsHL6 module was consistent with improved trichome growth in glumes. However, this expression pattern conflicted with the impaired trichome phenotype in OE leaves. The intrinsic mechanism behind this inconsistent phenomenon remains unclear in the current study.

Previous work showed that OsIPK2 stabilizes the Aux/IAA repressor OsIAA11 to block auxin signaling [34]. Thus, the trichome-promoting effect mediated by the OsWOX3B-OsHL6 module might be suppressed in leaves by *OsIPK2*-mediated auxin repression. Further, *OsIPK2* also represses gibberellin (GA) signaling, a pathway that generally promotes trichome growth [33]. Combined with these data, *OsIPK2* may integrate auxin and GA signaling to manipulate the core trichome regulatory cascade, thereby resulting in distinct trichome phenotypes between leaves and glumes. Future hormone rescue assays combined with genetic interaction analysis will provide more evidence to clarify the regulatory mechanism of *OsIPK2*-mediated organ-specific trichome development in the future.

### 3.2. Expression of OsIPK2 Inhibits Trichome Formation by Repressing the MBW Complex in Arabidopsis

A recent study showed that loss-of-function mutation of *AXR1*, a key upstream component of canonical auxin signaling, led to reduced trichome branching and altered trichome morphogenesis in *Arabidopsis*, while auxin overproduction in *YUCCA2*-overexpressing lines similarly reduces branch number [42]. These findings suggest that auxin acts as a positive regulator of trichome initiation and branching. In our study, *35S-OsIPK2* transgenic lines exhibited severely suppressed trichome initiation and branching, along with impaired auxin signaling in *Arabidopsis* (Figure 6 and Supplemental Figure S7). However, exogenous auxin treatment failed to rescue the trichome-defective phenotype of *35S-OsIPK2* plants (Supplemental Figure S8). Instead, transcription of core MYB–bHLH–WD40 (MBW) activator complex genes, including *GL1*, *GL2*, *GL3*, *EGL3*, and *TTG1*, was significantly downregulated in *35S-OsIPK2* lines (Figure 7). Thus, *OsIPK2* suppressed *Arabidopsis* trichome development primarily by repressing the MBW complex, rather than by perturbing auxin signaling. These results indicate that *OsIPK2* functions as a signaling regulator and acts upstream of the MBW complex to regulate the transcription of MBW genes in *Arabidopsis*. Since relevant protein–protein interaction and chromatin-level assays were not performed in this study, it remains unclear whether OsIPK2 directly interacts with MBW components. The detailed regulatory mechanism still requires further verification in future studies.

OsIPK2 and AtIPK2β are evolutionarily conserved inositol polyphosphate kinases that catalyze the conversion of IP_3_ to IP_4_ and further to IP_5_. Notably, overexpression of its *Arabidopsis* homolog *AtIPK2*β did not induce trichome defects (Supplemental Figure S9), suggesting that *OsIPK2* has undergone functional divergence from its *Arabidopsis* orthologs during evolution. In rice, OsIPK2 physically interacts with OsIAA11 to modulate auxin signaling and targets the OsSPL10-OsWOX3B-OsHL6 cascade that governs trichome development [34]. In contrast, AtIPK2β does not interact with Aux/IAA proteins or trichome-related regulators. Instead, it participates in flowering time control, ABA signaling, glucose responses, and chromatin modification by interacting with FVE, HDA6, SnRK1.1, and CPK4 [38,43,44]. When ectopically expressed in *Arabidopsis*, *OsIPK2* bypassed the endogenous *AtIPK2β* network and repressed transcription of the MBW complex, leading to impaired trichome initiation and branching. Thus, the observed functional divergence may arise from distinct protein–protein interactions and a downstream transcriptional cascade. The detailed regulatory mechanism and genetic interactions remain to be further verified in future investigations.

### 3.3. OsIPK2 Overexpression Affected Cuticular Wax Metabolism in Transgenic Rice

Beyond the transcriptional regulatory networks governing trichome development, our targeted wax metabolic profiling revealed distinct changes in fatty acid composition in *OsIPK2*-overexpressing rice leaves. Nonanoic acid (Pelargonic acid) was significantly downregulated in the OE-1 line relative to WT, whereas (E)-hexadec-9-enoic acid (Palmitoleic acid) was significantly upregulated (Figure 3). This shift indicates a redirection of metabolic flux from medium-chain fatty acid synthesis to the production of long-chain wax precursors, a metabolic alteration associated with the organ-specific trichome phenotypes observed in *OsIPK2* transgenic rice. Nonanoic acid is known to exert phytotoxic effects on plants by interfering with auxin transport, leading to inhibited root growth, abnormal morphological development, and altered cell ultrastructure [45,46]. (E)-hexadec-9-enoic acid is widely found in plant oils, and its accumulation in OE-1 line likely contributed to the remodeling of cuticular wax components in rice epidermal cells [47].

Our qPCR analysis further revealed that *OsGL1-1* was significantly downregulated in OE-1 and Ri-1 lines compared with WT (Figure 5). Previous studies have demonstrated that loss-of-function mutation of *OsGL1-1/WSL*2 led to a dramatic reduction in C22–C32 long-chain fatty acids, decreased cuticular wax deposition, and defective cuticle membrane in rice [48,49]. These data indicate that *OsGL1-1* is a potential downstream target of *OsIPK2*, and *OsIPK2* modulates rice cuticular wax metabolism at least in part by regulating the expression of *OsGL1-1*.

In *Arabidopsis*, cuticular wax metabolism is tightly coupled with trichome development. Both wax-deficient mutants and wax-overaccumulating lines trigger signaling cascades that alter trichome formation. For instance, *fdh*, *cer3*, *cer10*, and *abcg11* mutants show reduced wax and cutin levels, accompanied by decreased trichome density, smaller trichome size, and misshapen or collapsed trichome structures [20]. Conversely, increased wax accumulation in *bdg* mutant and *SHN* overexpression lines is also associated with cell wall defects, abnormal trichome morphology, and reduced trichome numbers [21,50]. It has been proposed that wax monomers indirectly inhibit trichome development in *Arabidopsis* by repressing *AtIPT3* expression and subsequent cytokinin biosynthesis, yet such a regulatory role of wax monomers in trichome development has not been reported in rice [51]. Our study identified a potential correlation between *OsIPK2* overexpression-mediated wax metabolic alterations and trichome development in rice. Based on these findings, we speculate that the *OsIPK2*-induced alterations in nonanoic acid and (E)-hexadec-9-enoic acid may be associated with rice trichome development, though this hypothesis requires further experimental validation.

### 3.4. Overexpression of OsIPK2 Promoted the Accumulation of Monosaccharides

Metabolic assay showed that D-Psicofuranose, D-Allofuranose, and D-Glucose were all significantly higher in the OE-1 line. Correlation analysis further revealed a strong positive correlation between these soluble carbohydrates and the long-chain wax precursor (E)-hexadec-9-enoic acid (Supplemental Figure S3), suggesting that *OsIPK2* coordinates carbohydrate metabolism and wax biosynthesis to modulate epidermal development.

Changes in carbohydrate status, especially glucose, are known to reprogram trichome initiation and branching in other plant species. In *Arabidopsis*, glucose positively regulates trichome initiation, branching, and growth by activating the HXK1-ACS7-ethylene-EIN3 signaling pathway [1]. In cotton, MYB212 positively regulates SWEET12 to facilitate the transport of sucrose and glucose from the ovary to the fiber, providing essential carbon sources and energy for cotton fiber elongation, a unicellular trichome-like structure [52]. However, in our study, the increased glucose content in *OsIPK2*-overexpressing plants was associated with reduced trichome density and length in leaves, which contrasts with the positive regulatory roles of glucose in *Arabidopsis* and cotton. This discrepancy indicates that the function of glucose in trichome formation is dependent on plant species, genetic background, or trichome type, reflecting the complexity of carbohydrate-mediated developmental signaling networks.

Notably, there are no previous reports linking rare sugars such as D-psicofuranose and D-allofuranose with trichome development. The specific roles of these rare sugars in epidermal cell differentiation remain unclear, but their positive correlation with (E)-hexadec-9-enoic acid suggests they may act as metabolic signals or substrates to coordinate wax biosynthesis and trichome development. Further functional validation, such as exogenous application of D-psicofuranose and D-allofuranose or knockdown of their biosynthetic genes, will help clarify their contributions to *OsIPK2*-mediated trichome regulation.

## 4. Materials and Methods

### 4.1. Plant Materials and Growth Conditions

The wild-type (WT) rice of Zhonghua11 (*Oryza sativa* L. ssp. *japonica*) and *Arabidopsis* (*Columbia* ecotype) were used for physiological experiments. Transgenic rice lines used included *OsIPK2-Ov-1* (OE-1), *OsIPK2-Ov-2* (OE-2) [35] as well as *OsIPK2-Ri-1* (Ri-1), *OsIPK2-Ri-2* (Ri-2) [36]. Transgenic *Arabidopsis* lines employed included *35S-OsIPK2-1*, *35S-OsIPK2-2*, *35S-OsIPK2-3* [35] and *35S-AtIPK2β* [44].

For the rice trichome phenotype assay, rice plants were grown in the field during summer and in a greenhouse during winter.

For the *Arabidopsis* trichome phenotype assay, seeds were surface-sterilized in 75% ethanol for 5 min and washed with sterilized water five times. Following 3 d of stratification in the dark at 4 °C, these seeds were germinated and grown on 1/2 Murashige and Skoog (MS) medium (0.8% agar and 2% Suc, pH 5.8) at 25 °C with cycles of 16 h of light and 8 h of darkness to produce *Arabidopsis* seedlings. For seed production or soil experiment, the seeds were planted in soil and grown in a greenhouse.

Auxin-resistant mutants, trichome-defective mutants, and relevant transgenic *Arabidopsis* lines used in this study were obtained from the Nottingham Arabidopsis Stock Centre (NASC), including *gl1-1* (Stock id. N64), *gl3-1* (N66), *gl3-1 egl3* (N6516), *gl1 gl3 egl3/pGL3::GL3-YFP* (N70981), *ARF2-RNAi* (N24870), and *iaa17/axr3-1* (N57504). Seeds of the *iaa16* mutant were kindly provided by Dr. Lucia C. Strader (Washington University in St. Louis, MO, USA).

### 4.2. Microstructure Observation of Trichome

To characterize the microstructure of mature rice leaf blades and glumes of *OsIPK2*-overexpression transgenic lines (OEi-1 and OE-2), *OsIPK2*-RNAi transgenic lines (Ri-1 and Ri-2), and WT plants, samples were observed using a Zeiss FE-SEM G300 scanning electron microscope (Zeiss, Jena, Germany) after sputter-coating with gold. The leaf images of transgenic lines and mutants were photographed using an Olympus IX71 microscope with an Olympus DP72 CCD camera (Olympus, Tokyo, Japan). At least three independent biological replicates were performed for each genotype.

### 4.3. Wax Metabolomic Analysis

Wax metabolites were isolated from leaf tissues using dichloromethane, with tetradecane-d50 added as an internal standard. After concentration under nitrogen gas, dried extracts were derivatized using BSTFA (containing 1% TMCS) and pyridine at 70 °C for 60 min. Excess derivatization reagents were removed under gentle nitrogen flow at 60 °C, and the derivatives were reconstituted in hexane and filtered through a 0.22 μm membrane prior to injection. GC-MS analysis was performed using an Agilent 8890 gas chromatograph coupled to a 7000D mass spectrometer (Agilent, Santa Clara, CA, USA) equipped with a DB-5MS column, with helium as the carrier gas.

Leaf surface area was determined by scanning fully unfolded leaves with an Epson Perfection V850 Pro scanner (Epson, Suwa, Japan). Images were imported into ImageJ (Version 1.44p) to set pixel-to-millimeter conversion for area quantification, and total double-sided area was calculated as 2 × single-sided area. For each biological replicate, 3–5 individual leaves were measured, and their total double-sided areas were summed for subsequent wax content normalization.

For metabolomic data analysis, unsupervised principal component analysis (PCA) was performed with R (www.r-project.org accessed on 1 December 2025) after unit variance scaling. Hierarchical cluster analysis (HCA) and Pearson correlation coefficients among samples were calculated using the ComplexHeatmap package. Differential metabolites were identified using orthogonal projections to latent structure-discriminant analysis (OPLS-DA), with selection thresholds of variable importance in the projection (VIP) > 1 and absolute log_2_-transformed fold change (|log_2_FC|) ≥ 1.0. A 200-permutation test was applied to validate the OPLS-DA model (Supplemental Figure S11). The permutation plot showed that all randomly permuted models had lower Q^2^ values than the original model (Q^2^ = 0.535, *p* = 0.175), and the original model’s R^2^Y (0.997) was significantly higher than the permuted models (*p* = 0.095). These data confirm that the model was not overfit, with group separation reflecting true wax metabolic differences rather than random noise. All identified metabolites were annotated using the KEGG Compound database and subsequently mapped to KEGG pathways for functional enrichment interpretation.

### 4.4. RNA-Seq and Data Analysis

The RNA extraction and RNA-seq were carried out as previously described [53]. Briefly, total RNA was extracted from mature leaf blades of wild-type and OE-1 plants, with three biological replicates per sample. RNA integrity was verified using an Agilent 2100 Bioanalyzer (Agilent, Santa Clara, CA, USA). Sequencing libraries were constructed with the TruSeq RNA Kit and sequenced on an Illumina HiSeq 2000 platform. Clean reads were aligned to the rice reference genome (IRGSP-1.0) with HISAT2, and gene/transcript expression levels (FPKM) were quantified using StringTie. Differentially expressed genes (DEGs) were screened with thresholds of |log_2_(fold change)| ≥ 1.0 and FDR < 0.05. Identification of significantly (hypergeometric test; FDR < 0.01) enriched GO categories was done using the DAVID online tool and database for KEGG and GO analysis (https://david.ncifcrf.gov/, accessed on 5 December 2025).

### 4.5. Quantitative RT-PCR

Total RNA was extracted from plant tissues using the Plant Total RNA Isolation Kit (Luoyang Huiqing Biotechnology, Luoyang, China). The FastKing gDNA Dispelling RT SuperMix (TIANGEN BIOTECH, Beijing, China) was used to remove the genomic DNA from the RNA extractions and synthesize the first-strand cDNAs. qRT-PCR was carried out using RR820A-TB Green^®^ Premix Ex Taq™ II (TAKARA, Osaka,, Japan) on a StepOnePlus real-time PCR system (Applied Biosystems, Foster City, CA, USA) following the instructions. The *OsACT2*, *OsUBQ5*, or *AtACT8* was used as the reference gene to normalize the target gene expression, which was calculated using the relative quantification method (2^−ΔΔCT^). For semi-quantitative RT-PCR, cDNA templates were used for PCR amplification with gene-specific primers. *AtACT2* was used as the internal control. PCR products were separated by agarose gel electrophoresis and visualized via ethidium bromide staining. The primers used in these experiments are listed in Supplemental Table S1.

### 4.6. Quantification of Anthocyanin Contents

For anthocyanin measurement, 6–10 seedlings were soaked in 600 μL 1% HCl (*v*/*v*) diluted with methanol, acidified and incubated for 12 h at 4 °C. After addition of 400 μL ddH_2_O and 1 mL chloroform, the anthocyanin contents in the aqueous phase were measured at 530 nm and 657 nm and calculated using the equation (A530 − A657)/Fw.

### 4.7. Analysis of the GFP Signal

The *35S-OsIPK2/DR5-GFP* plants were obtained by crossing the *35S-OsIPK2-1* transgenic lines with the homozygous *DR5-GFP* line. Homozygous plants were identified by segregation analysis, comparison with the parental phenotypes and PCR-based genotyping in the T_3_ progeny. The GFP signal in roots of *35S-OsIPK2/DR5-GFP* and Col/*DR5-GFP* plants was observed and photographed using an Olympus IX71 microscope with an Olympus DP72 CCD camera.

### 4.8. Accession Numbers

Sequence data from this article can be found at the Rice Annotation Project (RAP) website (https://rapdb.dna.affrc.go.jp/index.html, accessed on 30 March 2026) or the Arabidopsis Information Resource (TAIR) website (https://www.arabidopsis.org/, accessed on 25 March 2026) under the following accession numbers: *OsIPK2* (LOC_Os02g32370), *OsSCR1* (LOC_Os11g03110), *OsSCR2* (LOC_Os12g02870), *OsWOX3B* (LOC_Os05g02730), *OsHL6* (LOC_Os06g44750), *OsSAUR11* (LOC_Os02g42990), *OsACT2* (LOC_Os11g06390), *OsUBQ5* (LOC_Os01g22490), *GL1* (AT3G27920), *GL2* (AT1G79840), *GL3* (AT5G41315), *EGL3* (AT1G63650), *TTG1* (AT5G24520), *ETC1* (AT1G01380), *ETC2* (AT2G30420), *TRY* (AT5G53200), *CPC* (AT2G46410), *TCL1* (AT2G30432), and *AtACT8* (AT1G49240).

## 5. Conclusions

In this study, we identify *OsIPK2* as an organ-specific modulator of rice trichome development. Overexpression of *OsIPK2* suppressed macrohair initiation and microhair elongation in rice leaf but promoted trichome formation on the glumes. These organ-specific phenotypes were associated with transcriptional changes in the OsSPL10-OsSCR1/2-OsWOX3B-OsHL6 regulatory cascade and altered cuticular wax metabolism. Heterologous overexpression of *OsIPK2* in *Arabidopsis* repressed trichome initiation and branching by targeting the MBW complex rather than perturbing auxin signaling, reflecting its functional divergence in trichome regulation between species. These findings uncover a role of *OsIPK2* in mediating epidermal cell fate determination, and provide new insights into the species- and organ-specific regulation of trichome development. Future studies will focus on identifying direct targets of *OsIPK2* and clarifying how it integrates into trichome regulatory networks.

## Figures and Tables

**Table 1:** Quantitative analysis of the trichome phenotype of wild-type (Col) and *35S-OsIPK2* transgenic lines.

Trichome Branch Length (μm)	Col	*35S-OsIPK2-1*	*35S-OsIPK2-2*	*35S-OsIPK2-3*
Branch 1	208.5 ± 39.94	185.9 ± 27.67 ^a^	166.4 ± 24.35 ^a^	180.8 ± 52.72 ^a^
Branch 2	207.1 ± 33.76	175.6 ± 22.03 ^a^	153.2 ± 39.52 ^a^	146.7 ± 36.80 ^a^
Branch 3	202.4 ± 46.24	ND	ND	ND
Interbranch Zone length	95.42 ± 18.52	129.5 ± 24.37 ^a^	110.7 ± 1.999 ^a^	99.67 ± 17.90
Maximum Diameter	50.33 ± 8.319	37.91 ± 3.254 ^a^	37.56 ± 6.891 ^a^	27.99 ± 9.674 ^a^

^a^ Significantly different from wild type. Mean value ± SD. *n* = 30. ND, not determined. All trichomes in 10-day-old *35S-OsIPK2* lines were two-branched.

## Data Availability

The original contributions presented in this study are included in the article/Supplementary Material. Further inquiries can be directed to the corresponding authors.

## Supplementary Materials

The following supporting information can be downloaded at: https://www.mdpi.com/article/10.3390/plants15091414/s1. Figure S1. Cross-section of the leaves of WT and *OsIPK2*-overexpressing transgenic rice plants (OE-1) at the tillering stage. Figure S2. Classification of the 92 identified differentially accumulated cuticular wax metabolites (DAMs). Figure S3. Correlation heatmap of core differentially abundant metabolites (DAMs) in wild-type (WT) and OE-1 leaves. Figure S4. Expression levels of trichome and wax-related genes in wild-type and OE-1 line. Figure S5. Cross-section of the leaves of 21 d old Col and *35S-OsIPK2* transgenic lines. Figure S6. Growth phenotypes of 5-week-old *35S-OsIPK2* transgenic *Arabidopsis* lines, auxin-resistant mutants, and *ARF2-RNAi* plants. Figure S7. *OsIPK2* attenuated auxin signaling in *Arabidopsis* roots. Figure S8. Exogenous auxin could not rescue the trichome defect in *35S-OsIPK2* lines. Figure S9. *35S-AtIPK2β* transgenic *Arabidopsis* leaves showed no trichome-defective phenotype. Figure S10. Semi-quantitative PCR analysis of trichome regulatory genes in Col and *35S-OsIPK2* transgenic lines. Figure S11. The 200-permutation test of the OPLS-DA model. Table S1. Sequences of primers used in this study. Table S2. Wax metabolic analysis of leaves from WT and *OsIPK2* overexpression (Ov) transgenic plants. Table S3. RNA-Seq analysis of WT and *OsIPK2*-overexpressing transgenic rice plants (OE-1).

## References

[1] Wei Y.T., Bao Q.X., Shi Y.N., Mu X.R., Wang Y.B., Jiang J.H., Yu F.H., Meng L.S. (2025). Trichome development of systemic developing leaves is regulated by a nutrient sensor–relay mechanism within mature leaves. Sci. Adv..

[2] Li W., Wu J., Weng S., Zhang D., Zhang Y., Shi C. (2010). Characterization and fine mapping of the glabrous leaf and hull mutants (*gl1*) in rice (*Oryza sativa* L.). Plant Cell Rep..

[3] Liu L., Wang Y., Cao W., Yang L., Zhang C., Yuan L., Wang D., Wang W., Zhang H., Schiefelbein J. (2024). TRANSPARENT TESTA GLABRA2 defines trichome cell shape by modulating actin cytoskeleton in *Arabidopsis thaliana*. Plant Physiol..

[4] Larkin J.C., Walker J.D., Bolognesi-Winfield A.C., Gray J.C., Walker A.R. (1999). Allele-specific interactions between *ttg* and *gl1* during trichome development in *Arabidopsis thaliana*. Genetics.

[5] Payne C.T., Zhang F., Lloyd A.M. (2000). *GL3* encodes a bHLH protein that regulates trichome development in arabidopsis through interaction with GL1 and TTG1. Genetics.

[6] Khosla A., Paper J.M., Boehler A.P., Bradley A.M., Neumann T.R., Schrick K. (2014). HD-Zip proteins GL2 and HDG11 have redundant functions in *Arabidopsis* trichomes, and GL2 activates a positive feedback loop via MYB23. Plant Cell.

[7] Hung F.Y., Chen J.H., Feng Y.R., Lai Y.C., Yang S., Wu K. (2020). *Arabidopsis* JMJ29 is involved in trichome development by regulating the core trichome initiation gene *GLABRA3*. Plant J..

[8] Xie W., Zhao Y., Deng X., Chen R., Qiang Z., García-Caparros P., Mao T., Qin T. (2024). GLABRA3-mediated trichome branching requires transcriptional repression of *MICROTUBULE-DESTABILIZING PROTEIN25*. Plant Physiol..

[9] Schellmann S., Hülskamp M., Uhrig J. (2007). Epidermal pattern formation in the root and shoot of *Arabidopsis*. Biochem. Soc. Trans..

[10] Nemie-Feyissa D., Olafsdottir S.M., Heidari B., Lillo C. (2014). Nitrogen depletion and small R3-MYB transcription factors affecting anthocyanin accumulation in *Arabidopsis* leaves. Phytochemistry.

[11] Vadde B.V.L., Challa K.R., Sunkara P., Hegde A.S., Nath U. (2019). The TCP4 transcription factor directly activates *TRICHOMELESS1* and *2* and suppresses trichome initiation. Plant Physiol..

[12] Gonzalez A., Mendenhall J., Huo Y., Lloyd A. (2009). TTG1 complex MYBs, MYB5 and TT2, control outer seed coat differentiation. Dev. Biol..

[13] Cappellini F., Marinelli A., Toccaceli M., Tonelli C., Petroni K. (2021). Anthocyanins: From mechanisms of regulation in plants to health Benefits in foods. Front. Plant Sci..

[14] Zhang H., Wu K., Wang Y., Peng Y., Hu F., Wen L., Han B., Qian Q., Teng S. (2012). A WUSCHEL-like homeobox gene, *OsWOX3B* responses to NUDA/GL-1 locus in rice. Rice.

[15] Li J., Tang B., Li Y., Li C., Guo M., Chen H., Han S., Li J., Lou Q., Sun W. (2021). Rice SPL10 positively regulates trichome development through expression of *HL6* and auxin-related genes. J. Integr. Plant Biol..

[16] An Y., Ma X., Luo T., Chen L., Luo J., Su M., Hou S. (2025). OsSCR coordinates with OsSPL10 and OsWOX3B to promote epidermal hair development in rice. J. Integr. Plant Biol..

[17] Lan T., Zheng Y., Su Z., Yu S., Song H., Zheng X., Lin G., Wu W. (2019). *OsSPL10*, a SBP-box gene, plays a dual role in salt tolerance and trichome formation in rice (*Oryza sativa* L.). G3.

[18] Li J., Yuan Y., Lu Z., Yang L., Gao R., Lu J., Li J., Xiong G. (2012). *Glabrous Rice 1,* encoding a homeodomain protein, regulates trichome development in rice. Rice.

[19] Sun W., Gao D., Xiong Y., Tang X., Xiao X., Wang C., Yu S. (2017). Hairy Leaf 6, an AP2/ERF transcription factor, interacts with OsWOX3B and regulates trichome formation in rice. Mol. Plant.

[20] Berhin A., Nawrath C., Hachez C. (2022). Subtle interplay between trichome development and cuticle formation in plants. New Phytol..

[21] Oshima Y., Shikata M., Koyama T., Ohtsubo N., Mitsuda N., Ohme-Takagi M. (2013). MIXTA-like transcription factors and WAX INDUCER1/SHINE1 coordinately regulate cuticle development in *Arabidopsis* and *Torenia fournieri*. Plant Cell.

[22] Zheng H., Rowland O., Kunst L. (2005). Disruptions of the *Arabidopsis* enoyl-CoA reductase gene reveal an essential role for very-long-chain fatty acid synthesis in cell expansion during plant morphogenesis. Plant Cell.

[23] Rowland O., Lee R., Franke R., Schreiber L., Kunst L. (2007). The *CER3* wax biosynthetic gene from *Arabidopsis thaliana* is allelic to *WAX2/YRE/FLP1*. FEBS Lett..

[24] Bird D., Beisson F., Brigham A., Shin J., Greer S., Jetter R., Kunst L., Wu X., Yephremov A., Samuels L. (2007). Characterization of *Arabidopsis* ABCG11/WBC11, an ATP binding cassette (ABC) transporter that is required for cuticular lipid secretion. Plant J..

[25] Beaudoin F., Wu X., Li F., Haslam R.P., Markham J.E., Zheng H., Napier J.A., Kunst L. (2009). Functional characterization of the *Arabidopsis β* -ketoacyl-coenzyme A reductase candidates of the fatty acid elongase. Plant Physiol..

[26] Yang Q., Yang X., Wang L., Zheng B., Cai Y., Ogutu C.O., Zhao L., Peng Q., Liao L., Zhao Y. (2022). Two *R2R3-MYB* genes cooperatively control trichome development and cuticular wax biosynthesis in *Prunus persica*. New Phytol..

[27] Nadakuduti S.S., Pollard M., Kosma D.K., Allen C., Ohlrogge J.B., Barry C.S. (2012). Pleiotropic phenotypes of the *sticky peel* mutant provide new insight into the role of *CUTIN DEFICIENT2* in epidermal cell function in Tomato. Plant Physiol..

[28] Zhou L., Ni E., Yang J., Zhou H., Liang H., Li J., Jiang D., Wang Z., Liu Z., Zhuang C. (2013). Rice OsGL1-6 is involved in leaf cuticular wax accumulation and drought resistance. PLoS ONE.

[29] Jian L., Kang K., Choi Y., Suh M.C., Paek N.C. (2022). Mutation of *OsMYB60* reduces rice resilience to drought stress by attenuating cuticular wax biosynthesis. Plant J..

[30] Tao Z., Zhu L., Li H., Sun B., Liu X., Li D., Hu W., Wang S., Miao X., Shi Z. (2024). ACL1-ROC4/5 complex reveals a common mechanism in rice response to brown planthopper infestation and drought. Nat. Commun..

[31] Yadav R., Liu G., Rana P., Pullagurla N.J., Qiu D., Jessen H.J., Laha D. (2025). Conservation of heat stress acclimation by the IPK2-type kinases that control the synthesis of the inositol pyrophosphate 4/6-InsP7 in land plants. PLoS Genet..

[32] Yang S., Fang G., Zhang A., Ruan B., Jiang H., Ding S., Liu C., Zhang Y., Jaha N., Hu P. (2020). Rice *EARLY SENESCENCE 2*, encoding an inositol polyphosphate kinase, is involved in leaf senescence. BMC Plant Biol..

[33] Chen Y., Wei Z., Yang Q., Sang S., Wang P. (2017). Rice inositol polyphosphate kinase gene (OsIPK2), a putative new player of gibberellic acid signaling, involves in modulation of shoot elongation and fertility. Plant Cell Tissue Organ Cult..

[34] Chen Y., Yang Q., Sang S., Wei Z., Wang P. (2017). Rice inositol polyphosphate kinase (OsIPK2) directly interacts with OsIAA11 to regulate lateral root formation. Plant Cell Physiol..

[35] Chen Y., Han J., Wang X., Chen X., Li Y., Yuan C., Dong J., Yang Q., Wang P. (2023). *OsIPK2*, a rice inositol polyphosphate kinase gene, is involved in phosphate homeostasis and root development. Plant Cell Physiol..

[36] Chen Y., Li Y., Sang S. (2026). *OsIPK2* regulates seed vigor by integrating IP6 biosynthesis, auxin signaling, and H3K27me3 deposition in japonica rice. Biology.

[37] Lewandowska M., Keyl A., Feussner I. (2020). Wax biosynthesis in response to danger: Its regulation upon abiotic and biotic stress. New Phytol..

[38] Sang S., Chen Y., Yang Q., Wang P. (2017). *Arabidopsis* inositol polyphosphate multikinase delays flowering time through mediating transcriptional activation of *FLOWERING LOCUS C*. J. Exp. Bot..

[39] Méndez-Vigo B., Arteaga N., Murillo-Sánchez A., Alba S., Alonso-Blanco C. (2024). The bHLH transcription factor gene *EGL3* accounts for the natural diversity in *Arabidopsis* fruit trichome pattern and morphology. Plant Physiol..

[40] Hilscher J., Schlötterer C., Hauser M.T. (2009). A single amino acid replacement in ETC2 shapes trichome patterning in natural *Arabidopsis* populations. Curr. Biol..

[41] Xie Y., Yu X., Jiang S., Xiao K., Wang Y., Li L., Wang F., He W., Cai Q., Xie H. (2020). OsGL6, a conserved AP2 domain protein, promotes leaf trichome initiation in rice. Biochem. Biophys. Res. Commun..

[42] Liu L., Niu L., Ji K., Wang Y., Zhang C., Pan M., Wang W., Schiefelbein J., Yu F., An L. (2023). AXR1 modulates trichome morphogenesis through mediating ROP2 stability in *Arabidopsis*. Plant J..

[43] Yang Q., Sang S., Chen Y., Wei Z., Wang P. (2018). The Role of *Arabidopsis* Inositol Polyphosphate Kinase AtIPK2β in Glucose Suppression of Seed Germination and Seedling Development. Plant Cell Physiol..

[44] Wang P., Yang Q., Sang S., Chen Y., Zhong Y., Wei Z. (2017). *Arabidopsis* inositol polyphosphate kinase AtIpk2β is phosphorylated by CPK4 and positively modulates ABA signaling. Biochem. Biophys. Res. Commun..

[45] Dombrowski J.E., Martin R.C. (2014). Green leaf volatiles, fire and nonanoic acid activate MAPkinases in the model grass species *Lolium temulentum*. BMC Res. Notes.

[46] López-González D., Muñoz Usero M., Hermida-Ramón J.M., Álvarez-Rodríguez S., Araniti F., Teijeira M., Verdeguer M., Sánchez-Moreiras A.M. (2024). Pelargonic acid’s interaction with the auxin transporter PIN1: A potential mechanism behind its phytotoxic effects on plant metabolism. Plant Sci..

[47] Fatima T., Snyder C.L., Schroeder W.R., Cram D., Datla R., Wishart D., Weselake R.J., Krishna P. (2012). Fatty acid composition of developing sea buckthorn (*Hippophae rhamnoides* L.) berry and the transcriptome of the mature seed. PLoS ONE.

[48] Islam M.A., Du H., Ning J., Ye H., Xiong L. (2009). Characterization of *Glossy1*-homologous genes in rice involved in leaf wax accumulation and drought resistance. Plant Mol. Biol..

[49] Mao B., Cheng Z., Lei C., Xu F., Gao S., Ren Y., Wang J., Zhang X., Wang J., Wu F. (2012). Wax crystal-sparse leaf2, a rice homologue of WAX2/GL1, is involved in synthesis of leaf cuticular wax. Planta.

[50] Kurdyukov S., Faust A., Nawrath C., Bär S., Voisin D., Efremova N., Franke R., Schreiber L., Saedler H., Métraux J.P. (2006). The Epidermis-Specific Extracellular BODYGUARD Controls Cuticle Development and Morphogenesis in *Arabidopsis*. Plant Cell.

[51] Nobusawa T., Okushima Y., Nagata N., Kojima M., Sakakibara H., Umeda M. (2013). Synthesis of very-long-chain fatty acids in the epidermis controls plant organ growth by restricting cell proliferation. PLoS Biol..

[52] Sun W., Gao Z., Wang J., Huang Y., Chen Y., Li J., Lv M., Wang J., Luo M., Zuo K. (2019). Cotton fiber elongation requires the transcription factor GhMYB212 to regulate sucrose transportation into expanding fibers. New Phytol..

[53] Yu S.X., Hu L.Q., Yang L.H., Zhang T., Dai R.B., Zhang Y.J., Xie Z.P., Lin W.H. (2023). RLI2 regulates *Arabidopsis* female gametophyte and embryo development by facilitating the assembly of the translational machinery. Cell Rep..

