# Supplementary material for: OsIPK2 Acts as an Organ-Specific Modulator of Rice Trichome Development by Coordinating Cuticular Wax Metabolism and Transcriptional Regulation
Source: Plants (Basel). 2026 May 6;15(9):1414. doi: 10.3390/plants15091414 (PMC13165412; doi:10.3390/plants15091414)
Supplement: Supplementary file 1 [file plants-15-01414-s001.zip › Supplemental Figures S1-S11.pdf]

Supplementary material for

OsIPK2 acts as an organ-specific modulator of rice trichome development by coordinating cuticular wax metabolism and transcriptional regulation

Yao Chen [1,]*, Zhiqun Li [1], Ninghan Shi [1], Mengyang Huang [1], Yonghui Li [1], Kongyang Wu [1], Yanwei Cheng [1], Xuhao Liu [1], and Sihong Sang [2,]*

1 College of Life Sciences, Luoyang Normal University, Luoyang 471934, China

2 School of Pharmacy, Guizhou University of Traditional Chinese Medicine, Guiyang 550025, China

* Correspondence: cyzwhdx2011@whu.edu.cn (Y.C.); sihongsang@hotmail.com (S.S.)

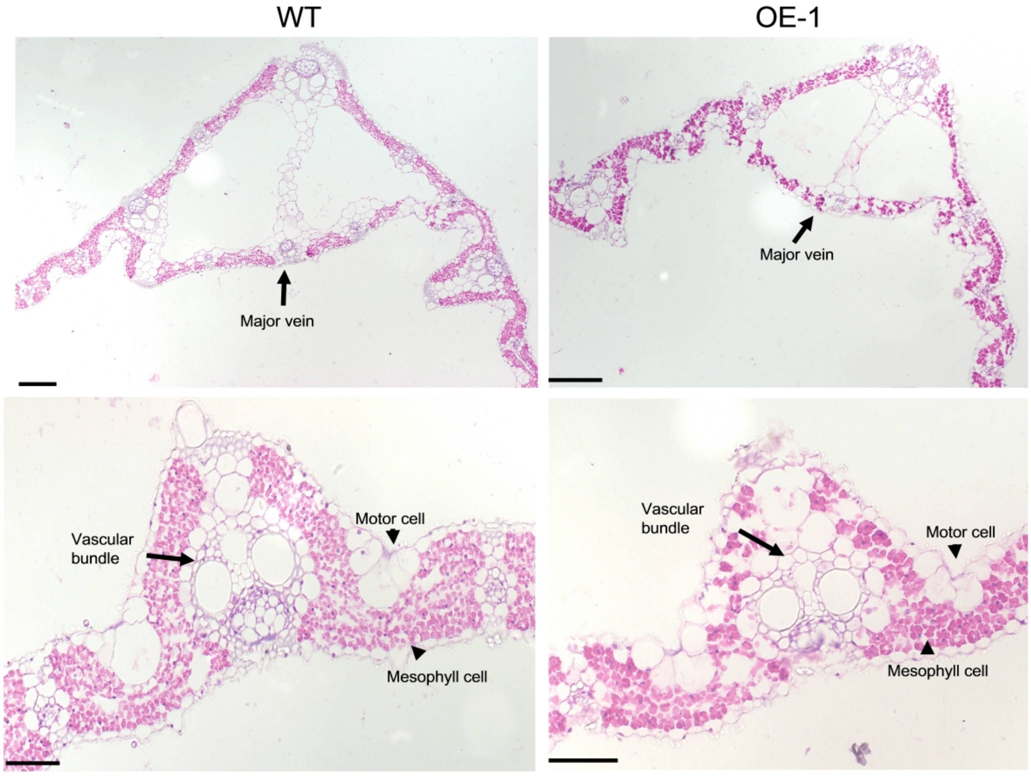

Figure S1. Cross-section of the leaves of WT and *OsIPK2*-overexpressing transgenic rice plants (OE-1) at the tillering stage. Scale bars= 100 μm.

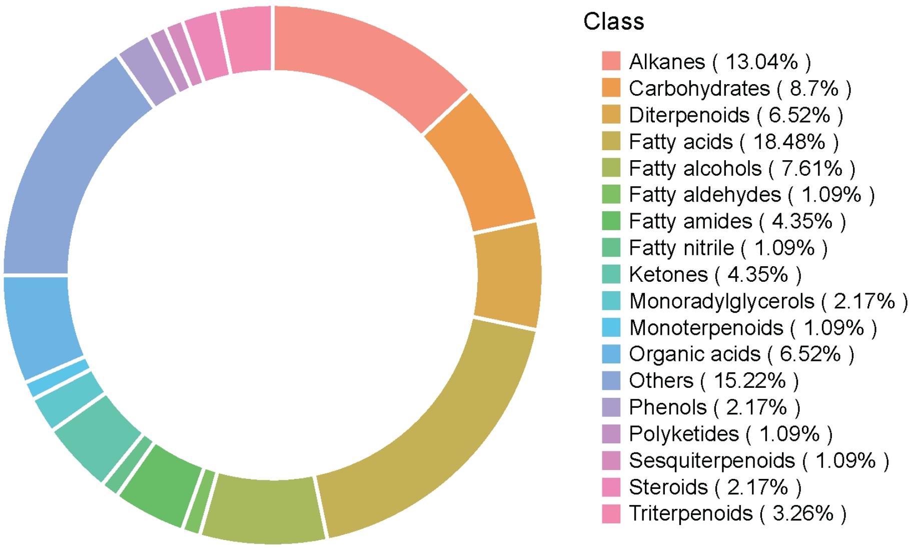

Figure S2. Classification of the 92 identified differentially accumulated cuticular wax metabolites (DAMs).

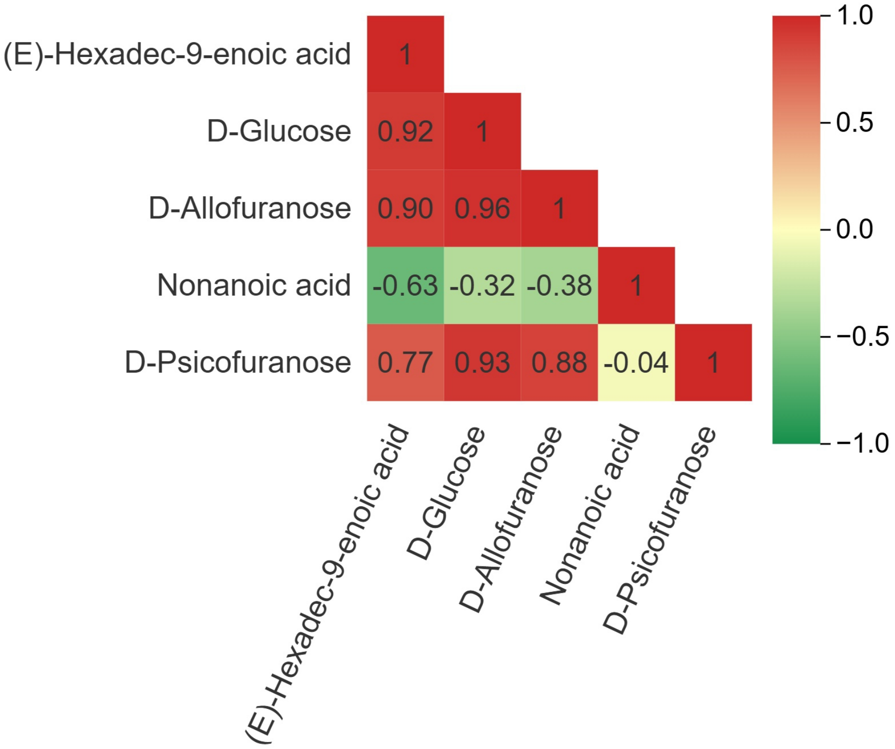

Figure S3. Correlation heatmap of core differentially abundant metabolites (DAMs) in wild-type (WT) and OE-1 leaves.

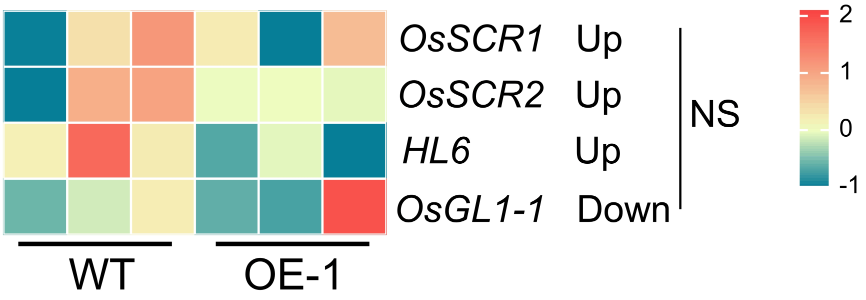

Figure S4. Expression levels of trichome and wax-related genes in wild-type and OE-1 line. NS, not significant.

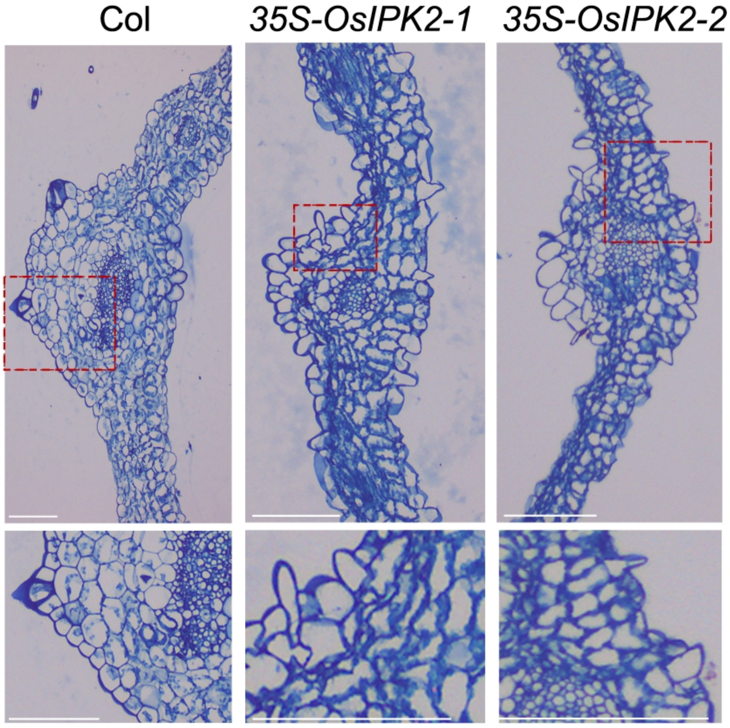

Figure S5. Cross-section of the leaves of 21-d-old Col and *35S-OsIPK2* transgenic lines. Scale bars= 2 mm.

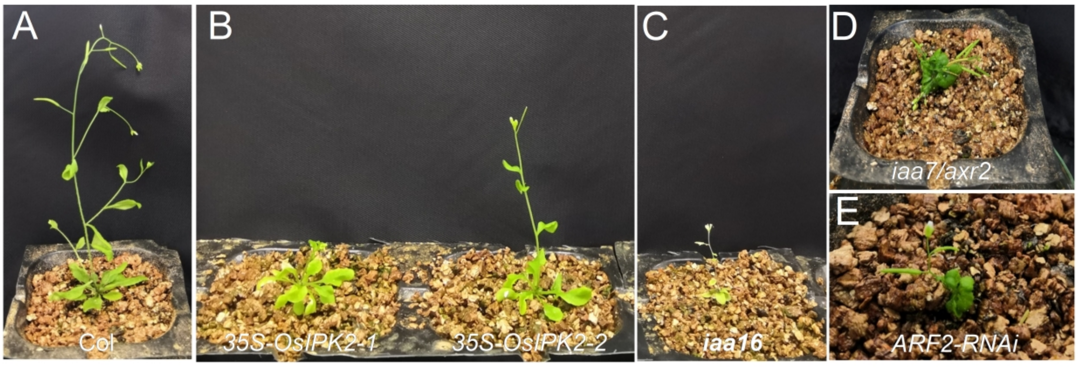

Figure S6. Growth phenotypes of 5-week-old *35S-OsIPK2* transgenic Arabidopsis lines, auxin-resistant mutants, and *ARF2-RNAi* plants. **A** Col-0 (Wild-type). **B** *35S-OsIPK2* lines. **C-D** Auxin-resistant mutants (*iaa16*, *iaa7/axr2*). **E** *ARF2-RNAi*.

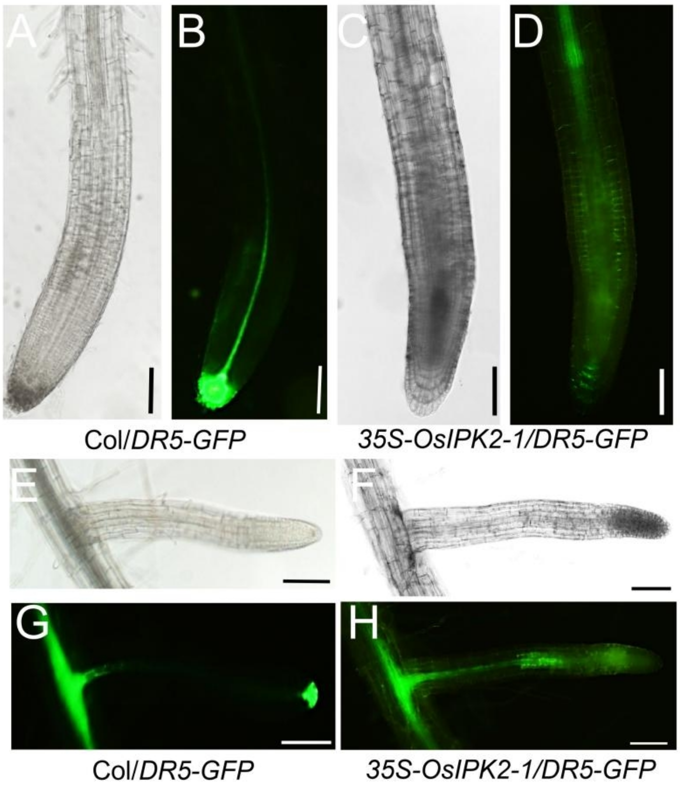

Figure S7. *OsIPK2* attenuated auxin signaling in Arabidopsis roots. **A-D** Bright-field (**A**, **C**) and corresponding *DR5-GFP* fluorescence (**B**, **D**) images of 5-day-old root tips of Col/*DR5-GFP* (**A, B**) and *35S-OsIPK2-1/DR5-GFP* (**C, D**) seedlings. **E-H** Bright-field (**E**, **F**) and DR5-GFP fluorescence (**G**, **H**) Lateral roots of 5-day-old Col/*DR5-GFP* (**E**, **G**) and *35S-OsIPK2-1/DR5-GFP* (**F**, **H**) seedlings. Scale bar = 100 μm.

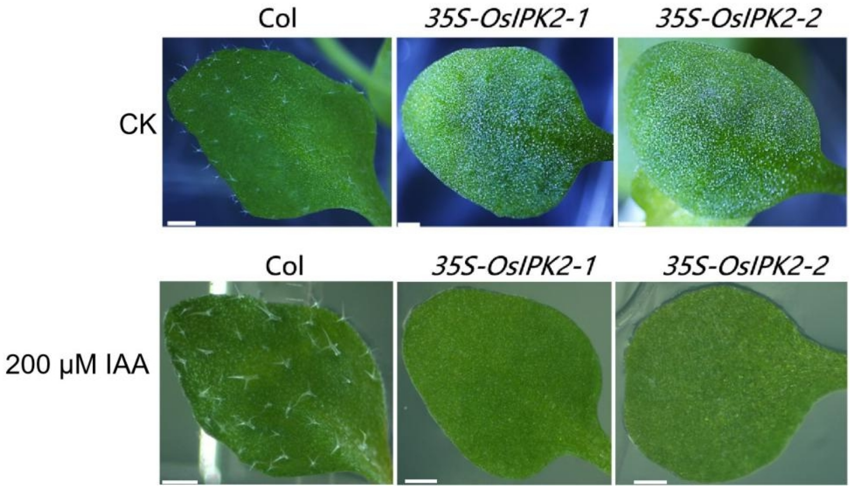

Figure S8. Exogenous auxin could not rescue the trichome defect in *35S-OsIPK2* lines. Trichome phenotypes of Col-0, *35S-OsIPK2-1*, and *35S-OsIPK2-2* leaves under control (CK, top) and 200 μM IAA-treated (bottom) conditions. Scale bar = 1 mm.

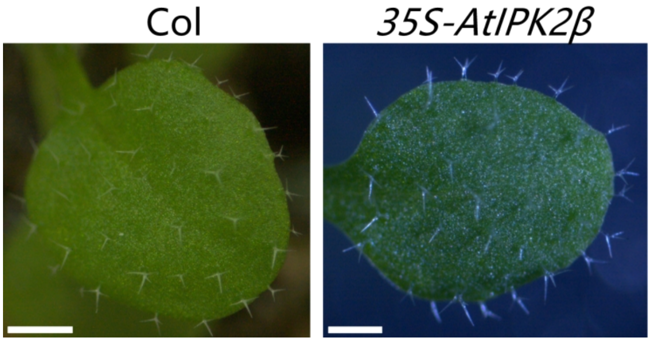

Figure S9. *35S-AtIPK2β* transgenic Arabidopsis leaves showed no trichome-defective phenotype. Scale bar = 1 mm.

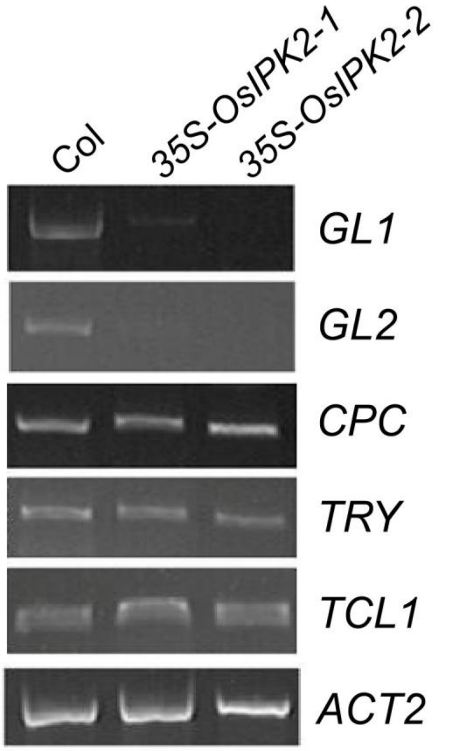

Figure S10. Semi-quantitative PCR analysis of trichome regulatory genes in Col and *35S-OsIPK2* transgenic lines. *AtACT2* was used as an control.

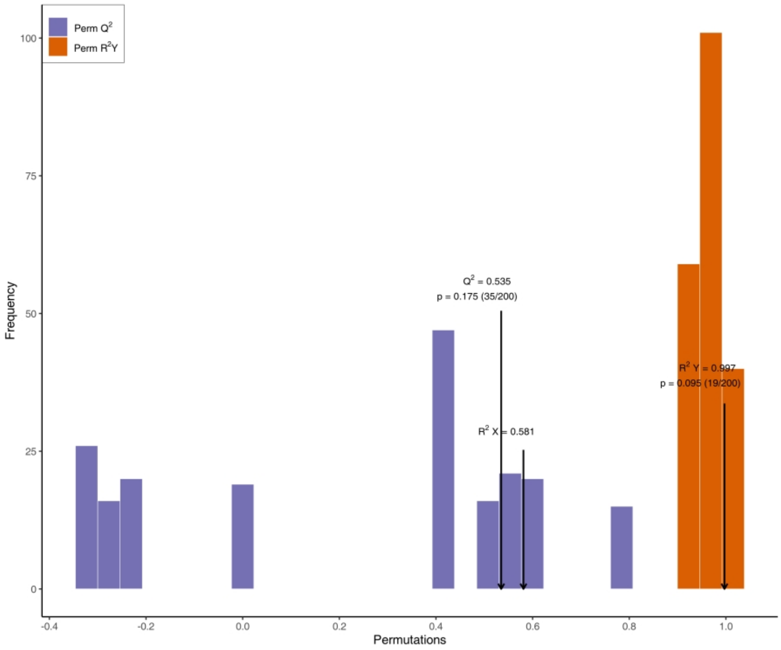

Figure S11. The 200-permutation test of OPLS-DA model.
